# Generation of dense statistical connectomes from sparse morphological data

**DOI:** 10.3389/fnana.2014.00129

**Published:** 2014-11-10

**Authors:** Robert Egger, Vincent J. Dercksen, Daniel Udvary, Hans-Christian Hege, Marcel Oberlaender

**Affiliations:** ^1^Computational Neuroanatomy Group, Max Planck Institute for Biological CyberneticsTuebingen, Germany; ^2^Graduate School of Neural Information Processing, University of TuebingenTuebingen, Germany; ^3^Bernstein Center for Computational NeuroscienceTuebingen, Germany; ^4^Department of Visual Data Analysis, Zuse Institute BerlinBerlin, Germany; ^5^Digital Neuroanatomy Group, Max Planck Florida Institute for NeuroscienceJupiter, FL, USA

**Keywords:** Peters' rule, barrel cortex, cortical column, thalamus, axon, dendrite

## Abstract

Sensory-evoked signal flow, at cellular and network levels, is primarily determined by the synaptic wiring of the underlying neuronal circuitry. Measurements of synaptic innervation, connection probabilities and subcellular organization of synaptic inputs are thus among the most active fields of research in contemporary neuroscience. Methods to measure these quantities range from electrophysiological recordings over reconstructions of dendrite-axon overlap at light-microscopic levels to dense circuit reconstructions of small volumes at electron-microscopic resolution. However, quantitative and complete measurements at subcellular resolution and mesoscopic scales to obtain all local and long-range synaptic in/outputs for any neuron within an entire brain region are beyond present methodological limits. Here, we present a novel concept, implemented within an interactive software environment called *NeuroNet*, which allows (i) integration of sparsely sampled (sub)cellular morphological data into an accurate anatomical reference frame of the brain region(s) of interest, (ii) up-scaling to generate an average dense model of the neuronal circuitry within the respective brain region(s) and (iii) statistical measurements of synaptic innervation between all neurons within the model. We illustrate our approach by generating a dense average model of the entire rat vibrissal cortex, providing the required anatomical data, and illustrate how to measure synaptic innervation statistically. Comparing our results with data from paired recordings *in vitro* and *in vivo*, as well as with reconstructions of synaptic contact sites at light- and electron-microscopic levels, we find that our *in silico* measurements are in line with previous results.

## Introduction

One of the major challenges in neuroscience is to relate results from structural and functional measurements across multiple spatial scales. Current anatomical approaches either provide information of synaptic connectivity at macroscopic, i.e., between brain regions (e.g., using bulk injections of retro/anterograde agents, Oh et al., 2014), mesoscopic, i.e., between cell types (e.g., using transgenic animal models, Wickersham et al., 2007), microscopic, i.e., between small numbers of individual neurons (e.g., using multi-electrode recordings in acute brain slices *in vitro*, Feldmeyer et al., 1999; Perin et al., 2011) or nanoscopic scales, i.e., reconstructing synaptic contact sites within small volumes (e.g., using electron microscopy in dense, Briggman et al., 2011, or sparsely labeled tissue, Schoonover et al., 2014). While all of these approaches provided important structural information about the neuronal circuitry, results obtained at different scales (and often even at the same scale when obtained by different methods) are largely incompatible. This prevents from generating wiring diagrams that provide quantitative and complete information of the number and subcellular location of all synaptic in/outputs for any neuron within and across brain areas (commonly referred to as “dense connectome”).

At present, methods that allow for measurements of synaptic connectivity at sufficiently high resolution (i.e., (sub)cellular levels) can be grouped into three main categories: First, electrophysiological approaches determine connectivity between pairs (or small numbers) of neurons using simultaneous patch-clamp recordings (e.g., Feldmeyer et al., 1999; Lefort et al., 2009), or combinations of single neuron recordings with optical stimulation, such as glutamate uncaging (Callaway and Katz, 1993; Schubert et al., 2007) or channelrhodopsin-assisted circuit mapping (Petreanu et al., 2009). Often, these approaches are combined with labeling the recorded neurons, allowing for reconstruction of the respective soma locations, dendrite morphologies and putative contact sites at light-microscopic levels (Feldmeyer et al., 1999, 2002; Sun et al., 2006; Frick et al., 2008; da Costa and Martin, 2011). Paired recording/reconstruction approaches are however limited to acute brain slices *in vitro*, where slice thicknesses of 300 μm result in substantial cutting of dendrites (Oberlaender et al., 2012a) and axons (Oberlaender et al., 2011), limiting these measurements to close-by neurons.

Second, electron-microscopic approaches, such as serial block face scanning (SBFSEM, Denk and Horstmann, 2004) or ion-beam techniques (Merchan-Perez et al., 2009), allow for automated imaging of small tissue volumes containing sparse (Lang et al., 2011) or densely labeled (Briggman et al., 2011) neuronal structures. Whereas technical issues of these microscope systems, which currently prevent from imaging larger volumes (e.g., an entire cortical column), may be overcome in the near future (Mikula et al., 2012), annotation and reconstruction of the rapidly increasing image data renders as the major bottleneck, limiting these approaches to tissue samples of at most 0.5 × 0.5 × 0.5 mm^3^ (Helmstaedter, 2013). Despite great progress in automated tracing (Kim et al., 2014), crowd sourcing of manual annotation (Helmstaedter et al., 2011) and combinations of manual and automated tools (Takemura et al., 2013), generation of complete dense connectomes (i.e., wiring diagrams that specify all in/outputs to a neuron) will require reconstructions of entire brain areas, spanning volumes of several cubic millimeters to centimeters, spatial scales that are multiple orders of magnitude beyond the present limits of these techniques.

Third, statistical approaches allow to determine cell type- and/or location-specific connectivity patterns by measuring structural overlap between reconstructed axons and dendrites of individual (Lubke et al., 2003) or bulk-labeled neurons (Meyer et al., 2010). Such approaches are commonly referred to as application of Peters' rule (White, 1979), but the validity of predicting synaptic connectivity by axo-dendritic overlap remains controversial (Mishchenko et al., 2010). The primary reason for this controversy arises from the fact that to date a quantitative and coherent framework to measure structural overlap is missing. Specifically, Peters' rule is often misinterpreted in a binary fashion, namely if dendrites and axons of two neurons overlap within a certain volume, it is assumed they are connected (Brown and Hestrin, 2009). In contrast, if dendrites and axons do not overlap, there will be no contact, the strongest implication from this approach. However, independent of the spatial scale at which the overlap is measured, within the respective overlap volume, dendrites and axons from other (unstained) neurons will be present and are equally likely to be connected to the stained neurons. Thus, overlap can never be assumed as evidence for a connection, but has to be interpreted as a probability for a connection with respect to all present neurons instead.

**Figure 1:**
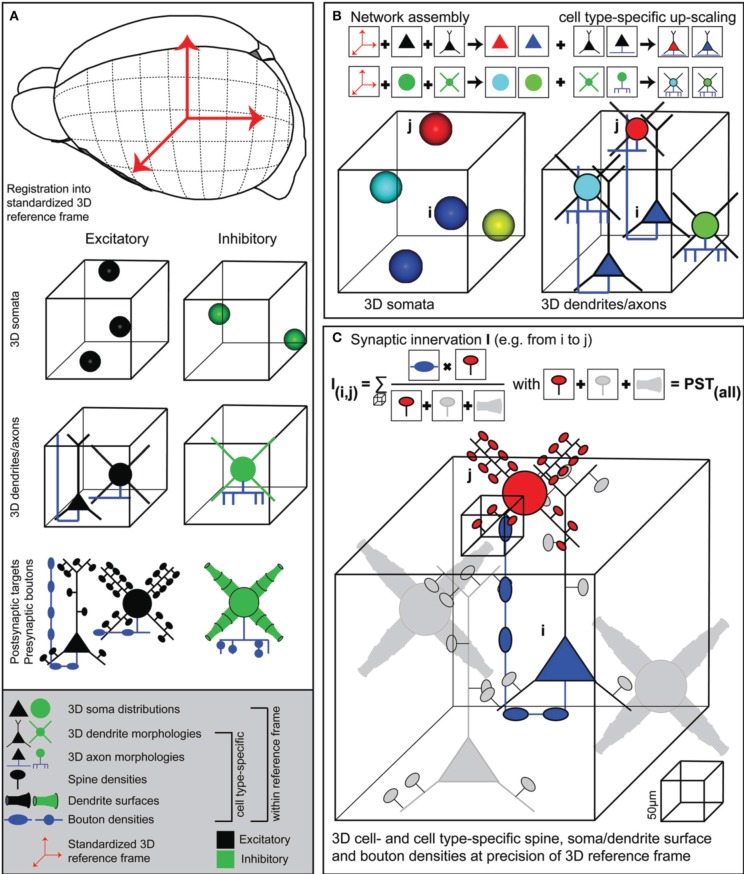
**Generating dense statistical connectomes**. **(A)** Generating a dense statistical connectome of a brain or brain region requires a standardized 3D reference frame of this brain region. The reference frame is used to register all anatomical data obtained from different experiments to a common coordinate system. Anatomical data to be collected from the brain region of interest: Number and 3D distribution of excitatory and inhibitory neuron somata; 3D reconstructions of representative samples of dendrites and axons of excitatory and inhibitory neuron cell types; determination of postsynaptic target densities, e.g., spine densities and dendrite surfaces, and presynaptic bouton densities for excitatory and inhibitory neuron cell types. **(B)** Anatomical data are assembled into a complete 3D network model. First, based on their 3D location, excitatory and inhibitory neuron somata are assigned to different anatomical substructures of the brain regions and to cell types. Next, somata of all cell types are replaced with dendrite and axon morphologies of the respective cell types. **(C)** Innervation from neuron *i* to neuron *j* is computed in 3D at a resolution determined by the anatomical variability of the 3D reference frame. This computation takes all possible postsynaptic targets of neuron *i* in addition to neuron *j* into account.

Here, we present a novel approach, implemented within an interactive software environment called *NeuroNet (NN)*, which formulates a coherent framework to measure structural overlap between two neurons, yielding connection probabilities with respect to all neurons present in the overlapping volume. This quantitative version of Peters' rule requires generation of an average dense model of the neuronal circuitry; dense referring to the fact that every neuron within the model of the brain structure of interest (i) has to be distributed according to measured 3D soma distributions, (ii) is represented by a complete 3D reconstruction of soma/dendrites/axon found at the respective location and (iii) contains information of cell type, as well as subcellular distributions of dendritic spines, diameters and axonal boutons (Figure 1A). *NN* allows integrating such anatomical data into a common reference frame that describes the average geometry, as well as its variability across animals, of the brain region(s) of interest (Figure 1B). Within the resolution of the reference frame, *NN* further allows to calculate synaptic innervation between any two neurons in the model, always taking all other neurons within the respective overlap volumes into account (Figure 1C). The resultant dense “statistical” connectome yields pairwise connection probabilities, numbers of putative synaptic contacts and subcellular synapse distributions for all neurons within an entire brain region, allowing for comparison of these *in silico* measurements with electrophysiological, light- and electron-microscopic data.

We illustrate our approach using the vibrissal part of rat primary somatosensory cortex (i.e., barrel cortex, vS1), present the required anatomical data and compare our *in silico* measurements of cell type-specific local (i.e., within a layer 4 (L4) barrel) and long-range (i.e., between thalamus and L4, L5, and L6 in vS1) innervation with previous results. Because our *in silico* measurements match previous *in vitro*/*vivo* data, we conclude that our concept of generating an average dense network model and providing a coherent framework to calculate Peters' rule in terms of innervation probabilities is an accurate alternative to any currently available connectivity mapping method. In addition, our approach now opens the possibility to investigate location-specific differences of connectivity within a population, as well as presence of higher-order connectivity patterns within and across cell types.

## Methods

### Design of *NeuroNet* software

The interactive software environment *NN* is implemented as an extension package for the *Amira* visualization software (FEI-Visualization Sciences Group, 2014), allowing for 3D visualization of anatomical input data, dense neuronal networks and synaptic connectivity measurements (Dercksen et al., 2012). *NN* comprises three major building blocks. First, the interface between *NN* and the anatomical input data is implemented as a *NeuralNetworkSpecification* data object. The user creates such a data object as a first step (initialized as an empty network) and loads all required input data (see specifications of data and format below). The *NeuralNetworkSpecification* object encapsulates all required anatomical data and can be saved to disk. Second, a network assembly module called *NeuronDistributor* takes the *NeuralNetworkSpecification* object as its input, integrates all anatomical data and performs an up-scaling operation to generate an average dense model of the network. The output of the *NeuronDistributor* module is a *SpatialGraphSet* data object, containing a list of transformed morphologies with an associated cell type. This *SpatialGraphSet* can be saved to disk. Third, a connectivity computation module called *NeuralNetworkAnalyzer* takes as input the *NeuralNetworkSpecification* and the *SpatialGraphSet* to calculate axo-dendritic overlaps between individual neurons. This compute module offers a query interface and selection/visualization options. The output generated by the *NeuralNetworkAnalyzer* includes a dense statistical connectome as represented by an innervation matrix *I_ij_* (for all selected neuron pairs *i* and *j*), as well as aggregate statistics about cell type- and location-specific connectivity, such as the convergence, divergence, connection probabilities, average number of synapses per cell or per cell type, and information about the number of neurons per cell type. These data can be saved as *AmiraMesh* tables or text files.

All routines of *NN* are implemented in C++ and the software is available for download online at http://www.zib.de/software/neuronet, including a manual for installation/usage and an exemplary dataset for testing the software. Downloads are available for Windows and Linux operating systems. *NN* supports multi-threaded computation using the OpenMP libraries. Computations presented in the Results section were performed on a desktop computer with 8 CPUs and 48 GB RAM. Hardware requirements depend on the size (number of neurons, dendritic/axonal lengths) of the neuronal network. For example, calculating connectivity between thalamus and all excitatory neurons within a single cortical column required memory of ~12 GB RAM. Instead, for networks containing several hundreds of thousands of neurons (e.g., for entire vS1), we recommend a compute-server with at least 64 CPUs and 500 GB RAM.

### Anatomical input data

Mandatory anatomical input data to *NN* comprise: 1. a standardized 3D reference frame, 2. 3D distributions of excitatory and inhibitory neuron somata, 3. representative samples of cell type-specific complete 3D morphological reconstructions and 4. measurements of cell type-specific subcellular distributions of soma/dendrite surface areas, dendritic spines and axonal boutons. In the following we introduce the formats for presenting the respective data to *NN*, provide example datasets for rat vS1 and review methodological approaches that allowed generating these example datasets (all anatomical data used in the Results section were acquired using experimental procedures carried out in accordance with the animal welfare guidelines of the Max Planck Society).

#### Standardized 3D reference frame

The most important prerequisite to assemble average dense models of the neuronal circuitry is the definition of a standardized 3D reference frame that allows integration of anatomical data obtained from many animals. In general, the reference frame describes the 3D geometry of the brain region(s) of interest in terms of anatomical landmarks. Further, it specifies the variability of these landmarks across animals, which serves as a resolution limit of the average circuit model. More specifically, the 3D reference frame has to describe (i) the boundaries of the brain region(s) of interest, (ii) anatomical substructures within these regions, and (iii) a global and/or multiple local coordinate systems. The latter reflects the general scenario that brain areas have irregular and/or curved boundaries and sub-structures.

**Figure 2:**
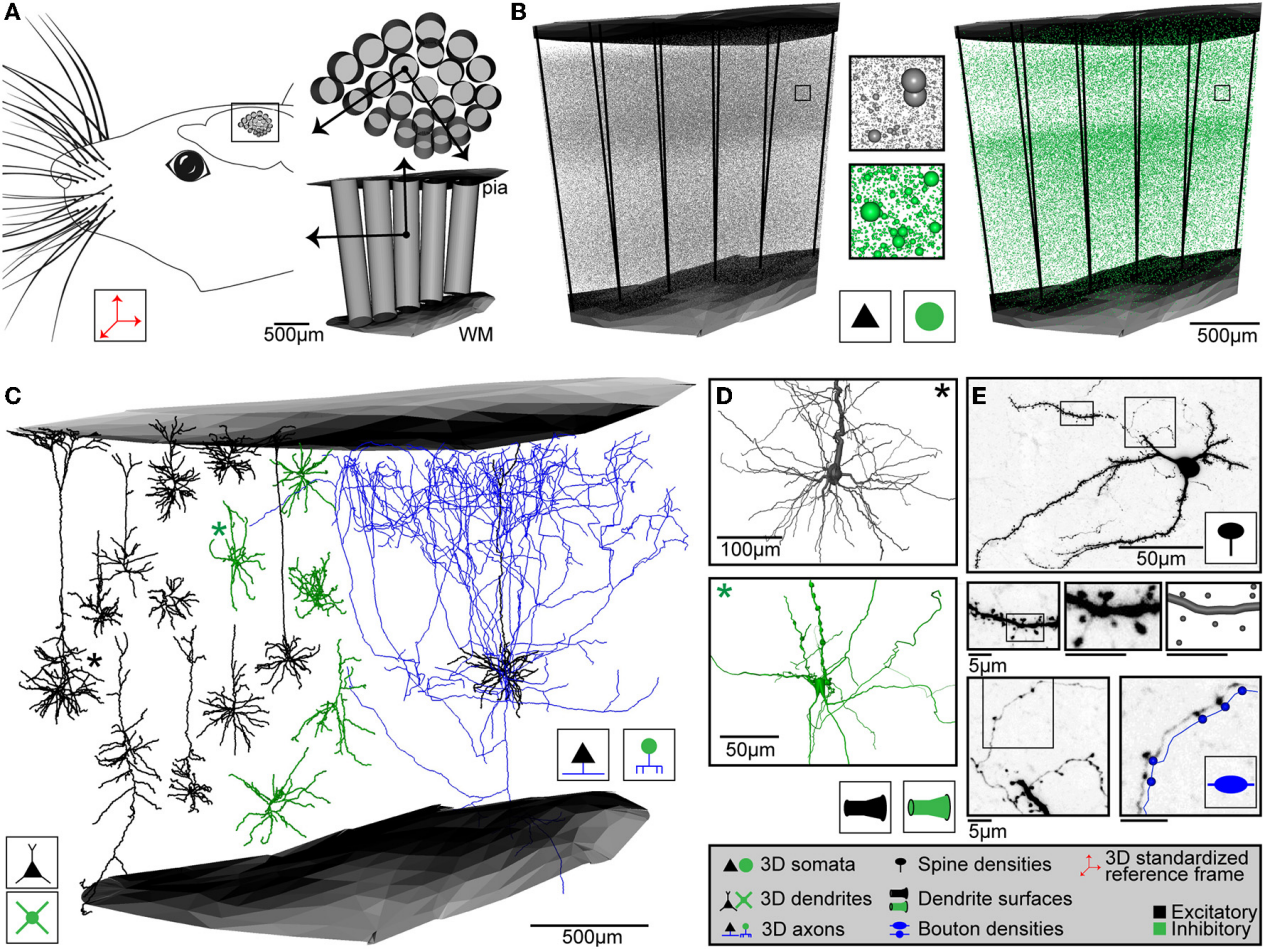
**Anatomical data used for generating dense statistical connectomes of rat vibrissal cortex (vS1)**. **(A)** Left: Rat vS1 contains segregated anatomical structures, called barrels, which are arranged somatotopically to the pattern of the large facial whiskers. Right top: Tangential view of barrels in the standardized rat vS1 cortex (see inset on left). These barrels provide natural landmarks for registration of anatomical data into the standardized reference frame. Bottom: Semi-coronal view of barrel columns in 3D. Pial and white matter (WM) surfaces delineate the vertical cortical boundaries in 3D. **(B)** 3D distribution of excitatory (left) and inhibitory (right) neuron somata with respect to cortical barrel columns in rat vS1. Center: Close-up view of neuron somata in insets in left and right panels. **(C)** Left: 3D dendrite reconstructions of 10 excitatory (black) and 5 inhibitory (green) cell types. Right: 3D dendrite (black) and axon (blue) reconstruction of an excitatory L5 slender-tufted pyramidal neuron. **(D)** Close-up views of the soma and dendrite surface reconstructions of an excitatory (black, top) and an inhibitory (green, bottom) neuron, corresponding to the dendrite morphologies marked with an asterisk (^*^) in **(C)**. **(E)** Determination of dendritic spines, dendrite surface and axonal boutons of a L4 spiny stellate neuron. Top: z-projection of a 50 μm thick section containing the soma, dendrites and axon branches. Center: From left to right: Close-up view of dendrite branch in left inset in top panel; close-up view of dendrite segment in inset in panel to the left; digital reconstruction of dendrite surface and spine locations of dendrite segment in panel to the left. Bottom left: Close-up view of axon branch in right inset in top panel. Bottom right: Close-up view of axon segment in inset in bottom left panel, with digital reconstruction of axon and bouton locations along the axon (shifted for visualization).

In case of rat vS1, the 3D reference frame has been generated by reconstructing the pial surface of entire rat cortex, the white matter tract (WM) and the circumferences of 24 cortical barrel columns (i.e., each representing one of the large facial whiskers on the animal's snout, Woolsey and Van der Loos, 1970), using high-resolution 3D images of the left hemisphere of Wistar rats at an age of 28 days (Egger et al., 2012). Repeating these reconstructions for 12 animals of the same strain and age, we superimposed all geometries using rigid transformations, minimized the distances between the respective center locations of the 24 barrel columns and calculated the average column center locations, column diameters and orientations, as well as the average 3D surfaces of the pia and WM above and below vS1, respectively (Figure 2A). The column centers are given with respect to a global coordinate system, where the z-axis is defined as the shortest perpendicular axis between the center of the barrel column representing the D2 whisker and the pial surface above the column. The x-axis points from the D2 center toward the center of the first adjacent rostral column (i.e., along the whisker row toward D3). The y-axis points approximately toward the first adjacent caudal column (i.e., along the whisker arc toward C2).

Because the pial and WM surfaces are curved, the orientation of each barrel column is tilted with respect to the (D2) z-axis. Therefore, we determined 23 additional local coordinate systems (i.e., for each barrel column), using the same approach used to determine the global D2 coordinate system. The final standardized reference frame of rat vS1 thus comprises the average pial and WM surfaces, 24 column center coordinates and diameters with respect to the global D2 coordinate system and 24 z-axes, representing local coordinate systems that define the orientation of each barrel column within the curved cortex. We further determined the variability of these anatomical landmarks across animals. The standard deviations (SDs) of the column center locations were on average ~90 μm, of the pia-WM distances ~100 μm and of the column orientations ~4.5 degrees (Egger et al., 2012). Thus, the geometry was remarkably preserved across animals and we defined the resolution limit of our average network model accordingly as 50 μm. Consequently, the volume comprising the standardized reference frame of rat vS1 was superimposed with a grid of 50 × 50 × 50 μm^3^ voxels and a local z-axis was calculated for each voxel by interpolating from the respective nearest barrel column axes.

The 3D reference frame of rat vS1 is presented to *NN* as follows: (1) A spreadsheet (*csv* file) contains information about the barrel column geometries with respect to the global coordinate system, i.e., the 3D center locations, column radii and a unit vector pointing along the respective orientation. Each column is further assigned a unique identifier (substructure) label. (2) A 3D vector field (*AmiraMesh vector field*) containing unit vectors at 50 μm resolution pointing toward the curved pial surface. In general, such vector fields should be sampled at the resolution of the 3D reference frame. (3) 3D boundary surfaces (*AmiraSurface* format) describing the 3D volume of the brain region (here: pial and WM surfaces). Additional boundary surfaces of anatomical substructures can be provided, e.g., borders of cytoarchitectonic cortical layers. *NN* currently supports the reference frame of vS1, but can be easily extended to other brain areas that can be described by 3D boundary surfaces and global and/or local coordinate systems. The resolution (i.e., voxel grid used for computations in *NN*, see below) can be adjusted to any value as determined by the inter-animal variability of the respective reference frame.

#### 3D soma distributions

The second anatomical prerequisite to generate an average dense model of the neuronal circuitry are measurements of the number and 3D distribution of excitatory and inhibitory neuron somata for the entire brain region(s) of interest. These distributions have to be obtained with respect to, and at the resolution of, the anatomical reference frame. In case of rat vS1, we stained 50 μm thick histological sections, cut tangentially to the D2 barrel column axis from the pia toward the WM, for NeuN (Mullen et al., 1992) and GAD67 (Kaufman et al., 1986; Kobayashi et al., 1987; Julien et al., 1990) to reveal all excitatory and inhibitory neurons, respectively. Using automated soma detection software (Oberlaender et al., 2009b), we determined the 3D center locations of all excitatory/inhibitory neuron somata for entire rat vS1 of four Wistar rats (age 28–29 days, Meyer et al., 2013, Figure 2B). For each counting dataset, we superimposed a 50 μm voxel grid and generated two 3D somata distributions for excitatory and inhibitory neurons, respectively (i.e., number of somata in 10^3^ per mm^3^). The two average soma density fields are provided to *NN* as 3D images (*AmiraMesh* format). We further determined the number of neurons per thalamic barreloid (Land et al., 1995; Meyer et al., 2013), which provide whisker-specific input to the respective barrel column (Brecht and Sakmann, 2002).

#### Cell type-specific 3D morphologies

The third prerequisite to generate an average dense model of the neuronal circuitry are reconstructions of complete 3D soma/dendrite/axon morphologies. The morphological dataset has to be representative for the brain region, fulfilling two criteria: (1) objective classification approaches should reveal all axo-dendritic cell types (i.e., dendrite as well as axon projection patterns are similar within, but significantly different between cell types) reported for the brain region(s) of interest (see, Narayanan et al., under review, for excitatory cell types in rat vS1), and (2) spatial sampling of neurons should be performed at the resolution of the anatomical reference frame (i.e., revealing location-dependent differences in morphology, spatial distribution and overlap of different cell types). For each cell type, a number of properties is defined using a spreadsheet (*csv* file) with predefined format: (1) whether the cell type is excitatory or inhibitory, (2) whether the morphology should be rotated during network assembly, i.e., if dendrites display asymmetric projections, such as polar dendrites pointing toward the center of a substructure (e.g., L4ss, Egger et al., 2008), (3) whether the reconstructions contain only axon or dendrites/axon, (4) whether the cell type has somata within and/or outside sub-structures (e.g., L4ss are only located inside the column, but not in septa between columns, Staiger et al., 2004; Bruno and Sakmann, 2006; Egger et al., 2008), and (5) the density of presynaptic contact sites (i.e., boutons) per μm axon, differentiated by sub-structures, in particular one value for boutons in infragranular, granular and supragranular layers of vS1, respectively. Finally, the spatial distribution of each cell type is determined by 3D boundary surfaces that describe the (sub)regions(s) where the cell type is found. If more than one cell type is present within such a 3D region, the relative frequency of morphologies from each cell type within the overlap region is specified using spreadsheets (*csv* files) with predefined format.

In case of rat vS1, we labeled individual neurons with Biocytin using cell-attached recordings *in vivo* (Pinault, 1996; Narayanan et al., 2014). After cutting the brain into 100 μm thick vibratome sections (i.e., tangential to the D2 barrel column axis, from the pia toward the WM), manual tracing software (e.g., *NeuroLucida*) or custom-designed semi-automated imaging and tracing systems (Oberlaender et al., 2007, 2009a; Dercksen et al., 2014) allow reconstruction of complete 3D morphologies with respect to the anatomical reference frame of rat vS1. Doing so, we reconstructed 153 excitatory neurons across the entire cortical depth (i.e., from L2 to L6) and used objective classification approaches to subdivide our sample into 10 axo-dendritic excitatory cell types (Figure 2C, Narayanan et al., under review). Because we obtained morphologies for every 50 μm of cortical depth, our spatial sampling is regarded as representative for rat vS1. Further, the 10 excitatory cell types represent all morphological classes that have been reported to date for rat vS1: L2 pyramids (L2, *n* = 16) and L3 pyramids (L3, *n* = 30) (Brecht et al., 2003; Staiger et al., 2014); L4 star pyramids (L4sp, *n* = 15), L4 spiny-stellates (L4ss, *n* = 22) and L4 pyramids (L4py, *n* = 7) (Staiger et al., 2004); L5 slender-tufted pyramids (L5st, *n* = 18) and L5 thick-tufted pyramids (L5tt, *n* = 16) (Hallman et al., 1988; Larkman and Mason, 1990); L6 corticocortical pyramids (L6cc, *n* = 11), L6 corticothalamic pyramids (L6ct, *n* = 13) and L6 inverted pyramids (L6inv, *n* = 5) (Kumar and Ohana, 2008). Consequently, sampling ~1% of all excitatory neurons located within a barrel column of rat vS1 is regarded as representative for all cell type-specific soma/dendrite/axon morphologies. Further, we reconstructed the cortical parts of thalamocortical axons (with respect to the reference structures of vS1, *n* = 14), labeled *in vivo* in the ventral posterior medial nucleus (VPM) of rat vibrissal thalamus (Oberlaender et al., 2012b). Similarly, axo-dendritic cell types of inhibitory interneurons (INH) need to be defined. Figure 2C illustrates five axo-dendritic INH types, as previously reported (Helmstaedter et al., 2009; Koelbl et al., 2013) and kindly provided by Moritz Helmstaedter, Dirk Feldmeyer and Hanno S. Meyer. At this point, it remains to be investigated whether these classes can be regarded as representative of rat vS1 in terms of the above stated criteria. INH morphologies are thus used purely for illustration of our approach throughout the present article. Further, in contrast to the excitatory dataset, INH morphologies were obtained by recording/labeling in acute brain slices *in vitro*. The total number of morphologies used in the subsequent application examples is 371 (153 excitatory and 204 inhibitory neurons from vS1 and 14 thalamocortical neurons from VPM).

*NN* expects these morphologies to be organized into folders according to [sub-structure label (e.g., barrel column ID)]/[cell type folder name]. The morphologies are specified either as *Amira SpatialGraphs* (Dercksen et al., 2014) or in the NEURON *hoc* language (Hines and Carnevale, 1997). If presented as *SpatialGraphs*, the branches comprising the morphologies have to be labeled as *Soma, ApicalDendrite, BasalDendrite*, or *Axon*, respectively. If specified in the *hoc* language, branches have to be labeled *soma, apical* for apical dendrites, *dendrite* for basal dendrites, or *axon*, respectively. Each cell type is represented twice, both as an axon cell type and a dendrite cell type. This implementation allows including long-range connections from cell types located in other brain regions (e.g., VPM axons, where soma/dendrites are located in the thalamus). The number of these long-range axon morphologies is specified in *NN* using a spreadsheet (*csv* file) with predefined format. In case of VPM axons, the number of morphologies innervating a respective barrel column is determined from cell counts in thalamus (i.e., the number of neurons per whisker-specific barreloid, Meyer et al., 2013).

#### Subcellular morphological statistics

The final anatomical prerequisite to generate an average dense model of the neuronal circuitry is measurements of the density of postsynaptic target sites (PSTs), i.e., spines along dendrites of excitatory neurons and surface areas of somata and dendrites of excitatory/inhibitory neurons for all cell types present within the brain region(s) of interest. 3D reconstruction of soma and dendrite diameters of excitatory and inhibitory neurons was performed manually using *NeuroLucida* software (Figure 2D). Dendritic spine densities and axonal bouton densities were determined manually from high-resolution 3D image stacks (92 × 92 × 200 nm^3^ voxel size) along skeleton tracings of *in vivo* labeled neurons of all cell types (Figure 2E). These data are grouped by morphological cell type.

Connections between cell types are specified in *NN* using a spreadsheet (*csv* file) with predefined format. For each possible connection between two cell types, the presynaptic cell type, postsynaptic cell type, as well as the normalized number of PSTs per μm^2^ area, and/or per μm branch length is defined, based on measured values (using the methods stated above) for each cell type and substructure (soma, apical dendrite, or basal dendrite). This meta-connectivity list thus specifies general knowledge of whether two cell types can in principle connect to each other and at which substructures. For example, inhibitory interneurons may specifically innervate somata and dendritic shafts of excitatory neurons. Thus, connections from interneuron to excitatory cell types can be specified in the meta-connectivity list such that PSTs are exclusively calculated by the surface areas of the excitatory somata and dendrites (i.e., soma/dendrite surface-specific PSTs). In contrast, connections from excitatory to excitatory cell types may be specified in the meta-connectivity list such that PSTs are calculated exclusively by the spine densities (i.e., dendrite length-specific PSTs).

### Data integration and up-scaling to generate average dense circuit models

**Figure 3:**
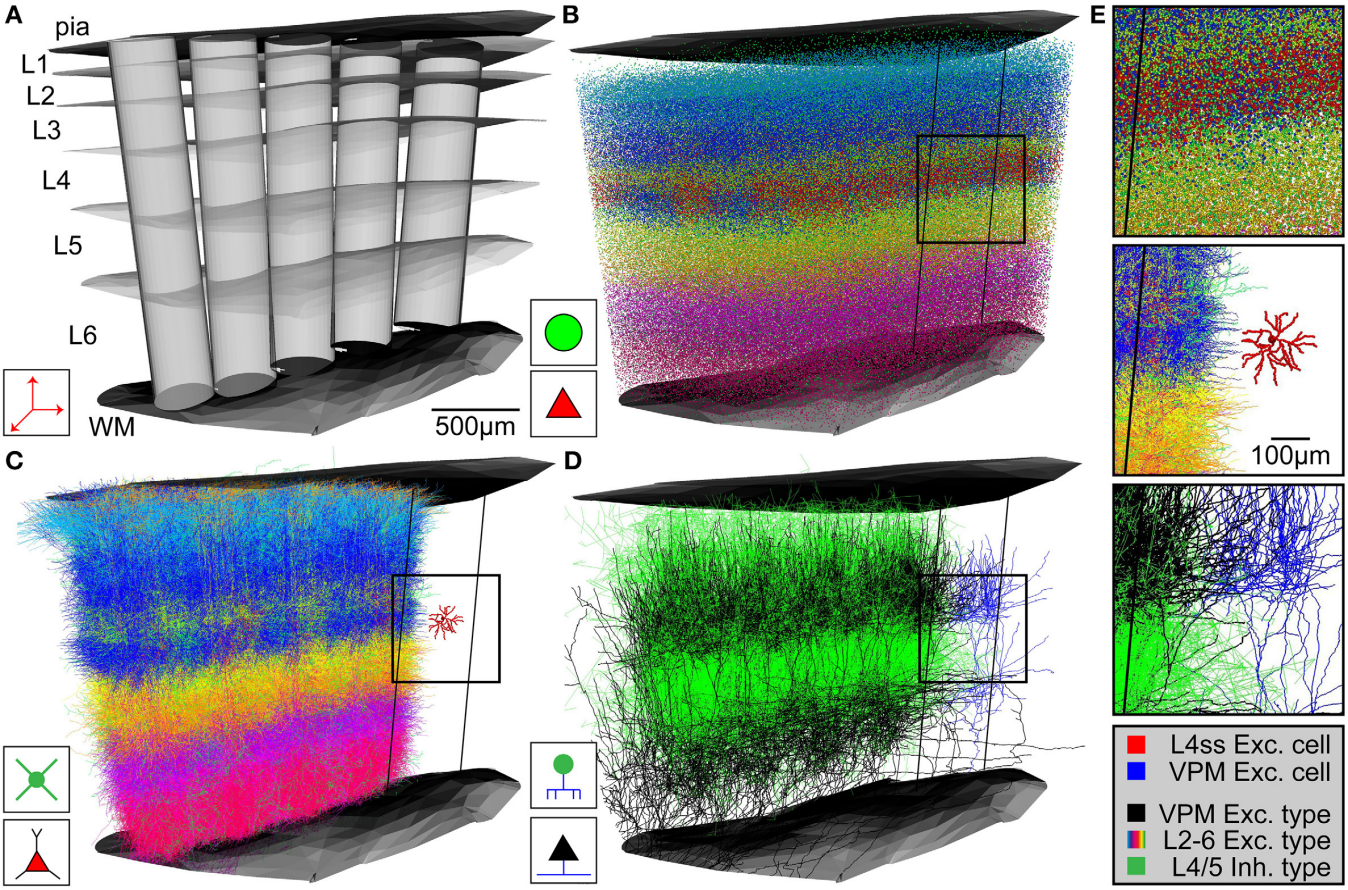
**Network assembly process**. **(A)** Standardized 3D reference frame of rat vibrissal cortex, with 3D organization of horizontal (i.e., barrel columns) and vertical (i.e., layers) structures. Every point in this brain region can be assigned to a barrel column and a cortical layer with 50 μm precision. **(B)** 3D distribution of 530,000 somata of 10 excitatory and 5 inhibitory cell types. **(C)** Replacement of somata with cell type-specific 3D dendrite morphologies. **(D)** Replacement of somata with cell type-specific 3D axon morphologies. Shown here: Thalamocortical axons from VPM (black), intracortical axons of inhibitory interneurons (green). **(E)** Top: Close-up view of inset in **(B)**. Center: Close-up view of inset in **(C)**, showing the dendrites of a single L4 spiny stellate (L4ss) neuron (red) next to all dendrites from all cell types in the neighboring barrel column. Bottom: Close-up view of inset in **(D)**, showing a single thalamocortical VPM axon (blue) next to all axons from two cell types in the neighboring barrel column.

Upon availability of the above described anatomical data in appropriate formats, *NN* automatically generates an average dense representation of the neuronal circuitry of the brain region defined by the reference frame (Figure 3). First, the cell type-specific boundary surfaces are integrated (Figure 3A shows a subsample of the cell type-boundaries) into the 3D reference frame. Next, the excitatory and inhibitory somata distributions are registered into the 3D reference frame. Excitatory and inhibitory soma positions are generated for all voxels in the soma density grid by multiplying the respective density values with the voxel volume (e.g., 50^3^ μm^3^) and rounding to the nearest integer. 3D soma locations within a voxel are drawn from a uniform distribution. Based on the 3D location, each soma is further assigned to a unique substructure (barrel column) and cell type (Figure 3B). Each soma is assigned to the barrel column (modeled as a cylinder) that is closest to the 3D soma position. To determine the cell type, first the region containing the soma is determined by identifying its location with respect to the cell type boundary surfaces. The cell type is then selected randomly based on the relative frequency of cell types within this region (as specified by the respective *csv* file). Soma/dendrite morphologies are then placed at all computed soma positions (Figure 3C). For each soma, a dendrite morphology is chosen at random from all morphologies fulfilling the following criteria: (1) the cell type of the morphology is the same as the cell type assigned to the soma, (2) the morphology is registered to the sub-structure (e.g., column) that is closest to the new soma location, and (3) the soma location of the morphology is not further away from the new soma location than one voxel of the reference frame resolution (i.e., in case of rat vS1, the original soma location of the morphology and its location within the model are within ± 50 μm along the z-axis of the respective column). The latter step guarantees that potential location-specific morphological properties are preserved within the resolution limit of the reference frame. Lastly, the morphologies are transformed as follows: (i) translation of the morphology to the new soma location; (ii) rotation around the soma, such that the vertical orientation is preserved and optionally (iii) cells with asymmetric projection patterns (e.g., polar dendrites) are rotated such that their orientation is retained (e.g., L4ss are rotated around the column axis to preserve projections toward the barrel column center). Third, axon morphologies of each cell type are inserted to match the number of somata/dendrites for each cell type (Figure 3D). For each soma, an axon morphology is chosen at random from all morphologies fulfilling the following criteria: (1) the cell type of the morphology is the same as the cell type assigned to the soma, and (2) the morphology is registered to the substructure (e.g., column) that is closest to the soma location. In contrast to dendrite morphologies, axon morphologies are not transformed to new soma locations to prevent that rotation/translation results in loss of location-specific projection patterns (e.g., L4ss neuron in vS1 display axons confined to the respective barrel column containing the soma (Egger et al., 2008) and hence translations would result in inappropriate innervation of septal areas). Long-range axons innervating the modeled brain region (i.e., their somata are located elsewhere) are registered in the same way as cell types with somata inside the brain region of interest, preserving their vertical and horizontal projection patterns with respect to the reference frame at 50 μm resolution. Then, long-range axons are up-scaled (i.e., duplicated) until the number of morphologies specified for this cell type (i.e., in the input *csv* file) is reached (e.g., VPM axons are up-scaled to meet the average number of somata per thalamic barreloid, e.g., 311 for the D2 whisker, Meyer et al., 2013). The result of the network assembly step is a dense representation of the neuronal circuitry of an entire brain region, where each neuron of a measured 3D soma distribution is represented by dendrite/axon morphologies of the appropriate cell type and location/orientation within the resolution of the geometrical reference frame (Figure 3E).

### Calculation of statistical synaptic innervation at subcellular levels

The dense statistical connectome *I_ij_* is computed as follows: First, for each presynaptic neuron *i* its axon is converted into a 3D bouton density at the resolution of the reference frame by clipping the axon of neuron *i* with all six faces of each voxel, summing up the length of the respective axon branches within the voxel and multiplying this value by the cell type- and substructure-specific bouton length density. Second, each postsynaptic neuron *j* is converted into a 3D PST density at the resolution of the reference frame by clipping the soma and dendrites of neuron *j* with all six faces of each voxel, summing up the length and the surface area of the respective dendrite branches and the soma and multiplying these values by the connection-specific PST length or area density. Dendrite and soma surface area are computed from the diameter values along the branches using trapezoidal integration. 3D PST densities of each postsynaptic neuron *j* for connections with neurons of cell type *T(i)* of the presynaptic neuron *i* in the voxel centered on $\vec{x}$ are determined as the sum of two terms *PST_spines_* + *PST_surface_*):

$$\begin{array}{l} PST_{j}\left(\vec{x},T(i)\right)=\sum_{labels\;L}l_{j,L}(\vec{x})\cdot \lambda_{T(i),T(j)}(L)\\ \;+\;\sum_{labels\;L}a_{j,L}(\vec{x})\cdot \alpha_{T(i),T(j)}(L) \end{array}$$

Here, *label L* refers to a subcellular structure of the postsynaptic neuron, i.e., soma, basal dendrite or apical dendrite. *l_j,L_*($\vec{x}$) is the total length of all compartments of label *L* of neuron *j* inside the voxel centered on $\vec{x}$ (in μm). λ_*T(i),T(j)*_(*L*) is the length PST density (e.g., 1 spine per μm basal dendrite) for connections from neurons of type *T(i)* to neurons of type *T(j)* onto target structures with label *L* (in μm^−1^), as provided by spine density measurements and specified in the meta-connectivity spreadsheet. *a_j,L_*($\vec{x}$) is the total surface area of all compartments of label *L* of neuron *j* inside the voxel centered on $\vec{x}$ (in μm^2^). α_*T(i),T(j)*_(*L*) is the surface PST density (e.g., 0.4 PSTs per μm^2^ soma surface) for connections from neurons of type *T(i)* to neurons of type *T(j)* onto target structures with label *L* (in μm^−2^). Whereas spine and bouton distributions can be measured (e.g., using the methods stated above), we derived surface PST densities by assuming that the total number of boutons *B_all_*($\vec{x}$) from all presynaptic cell types *T*(*i*) should match the number of total PSTs from all cell types *T*(*j*):

$$\sum_{i,j}PST_{surface,j}(\vec{x},T(i))=B_{all}(\vec{x})-PST_{spines}(\vec{x})$$

Reducing this equation to 1 dimension (i.e., collapsing the 3D densities to the z-axis), we fit the respective surface PST density values α_*T(i),T(j)*_ using standard least squares algorithms (see fitting result in the online meta-connectivity list).

Third, the precision (across animal variability) of the geometrical reference frame determines the voxel resolution, i.e., the smallest scale at which axo-dendritic overlap can be calculated between morphologies obtained in different animals. Thus, locations of somata/dendrites/axons within a voxel cannot be further resolved and proximity of boutons and PSTs within a voxel cannot be used to estimate synaptic innervation. Instead, we assume that all PSTs within a voxel are equally likely to receive any bouton in the same voxel (i.e., independent synapse formation at resolutions smaller than the accuracy of the reference frame). The probability that neuron *j* is targeted by a bouton within the voxel centered on $\vec{x}$ is then given by:

$$p_{j}(\vec{x},T(i))=\frac{PST_{j}(\vec{x},T(i))}{PST_{all}(\vec{x},T(i))}$$

Here, *PST_all_*($\vec{x}$, *T*(*i*)) refers to the total number of potential postsynaptic contact sites for connections with presynaptic cell of type *T(i)* in the voxel centered on $\vec{x}$, i.e.,

$$PST_{all}(\vec{x},T(i))=\;\sum_{j}PST_{j}(\vec{x},T(i))$$

If *B_i_* boutons from neuron *i* are present in the voxel at $\vec{x}$, the probability that neuron *j* is targeted by *n* of these boutons is given by the binomial distribution:

$$P(n;p_{j},B_{i})=\left(\begin{array}{c} B_{i}\\ n \end{array}\right)p_{j}^{n}{\left(1-p_{j}\right)}^{B_{i}-n}$$

Average values for *B_i_* and *p_j_* in our networks are *O*(10^1^)-*O*(10^2^) and *O*(10^−3^), respectively. Given the ~5 orders of magnitude differences between *B_i_* and *p_j_*, we can approximate the binomial distribution by a Poisson distribution (i.e., *B_i_* → ∞ and *p_j_*→ 0):

$$P\left(n;{\tilde{I}}_{ij}(\vec{x})\right)=\frac{{\tilde{I}}_{ij}^{n}(\vec{x})}{n!}\text{exp}(-{\tilde{I}}_{ij}(\vec{x}))$$

Here, we defined the average innervation *Ĩ_ij_*($\vec{x}$) from neuron *i* to neuron *j* in the voxel at $\vec{x}$:

$${\tilde{I}}_{ij}(\vec{x}):=B_{i}(\vec{x})\cdot p_{j}(\vec{x})$$

The connectivity statistics between any two neurons *(i,j)* can thus be described by the 3D scalar field *Ĩ_ij_*($\vec{x}$). The probability of finding a connection between any two neurons *i* and *j* within a specific voxel located at $\vec{x}$ is further given by:

$$p_{ij}(\vec{x})=1-P(n=0;{\tilde{I}}_{ij}(\vec{x}))=1-\text{exp}(-{\tilde{I}}_{ij}(\vec{x}))$$

Because we assume that synapses in different voxels are formed independently of another, the total probability of finding a connection between two neurons *i* and *j* is:

$$\begin{array}{l} p_{ij}=1-\prod_{\vec{x}}P(n=0;{\tilde{I}}_{ij}(\vec{x}))=1-\text{exp}(-\sum_{\vec{x}}{\tilde{I}}_{ij}(\vec{x}))\\ \;=1-\text{exp}(-I_{ij}) \end{array}$$

Here, $I_{ij}\text{ ​}:=\Sigma_{\vec{x}}{\tilde{I}}_{ij}(\vec{x})$ is the total (i.e., summed over all voxels) average innervation from neuron *i* to neuron *j*. Intuitively, *I_ij_* is the expected number of synapses connecting neuron *i* to neuron *j*.

### Calculation of statistical synaptic innervation at cell type levels

Using the innervation matrix *I_ij_* for all pairs of neurons in the network, analyses can be extended to the population level, allowing comparison with pairwise connectivity measurements performed *in vitro/vivo*. *In silico*, pairwise connectivity between two populations (pre: A and post: B) can be described by three experimentally accessible parameters: the convergence *C_b_*, i.e., the fraction of the presynaptic population connected to a single postsynaptic neuron *b* ϵ *B*, the divergence *D_a_*, i.e., the fraction of the postsynaptic population targeted by a single presynaptic neuron *a* ϵ *A*, and the connection probability *P_AB_*, i.e., the probability that any two neurons *a* ϵ *A, b* ϵ *B* are connected. We can now define these three quantities in terms of the neuron-to-neuron connection probability *p_ij_* = 1 − exp( − *I_ij_*) introduced above:

$$\begin{array}{l} C_{b}={\langle p_{ab}\rangle }_{a\in A}\\ D_{a}={\langle p_{ab}\rangle }_{b\in B}\\ P_{AB}={\langle p_{ab}\rangle }_{a\in A,b\in B} \end{array}$$

Here, 〈···〉_*a* ϵ *A*_ is the ensemble average across all neurons *a* in population *A* etc. Additionally, we can compute the distribution of the number of synapses per connection *n_AB_* between these two populations by averaging across the individual synapse number distributions *n_ij_* := *P*(*n*; *I_ij_*):

$$n_{AB}={\langle n_{ab}\rangle }_{a\in A,b\in B}={\langle Poisson(I_{ab})\rangle }_{a\in A,b\in B}$$

## Results

### Application example 1: dense 3D model of rat vS1

Based on the anatomical input data (Figure 2) specified in the Methods section, we used *NN* to generate an average dense model of entire rat vS1 (Figure 3). The model consists of 10 excitatory and 5 inhibitory axo-dendritic cell types, in 24 barrel columns. The total volume of the vS1 model was 6.4 mm^3^ (Egger et al., 2012).

First, the average 3D distributions of excitatory and inhibitory somata were registered to the reference frame and somata were placed and assigned to cell types (Figure 3B) and anatomical substructures as described above (i.e., each soma contains four labels: the nearest barrel column, whether the soma is inside the column or within the septum, the cell type, excitatory or inhibitory). The total number of neurons within the model was 529926, with 462436 being excitatory and 67490 being inhibitory. Neuron numbers and their 3D distributions are within the mean ± SD (529715 ± 39104) of the measured soma distributions at 50 μm resolution (Meyer et al., 2013). Next, *NN* replaced each soma by appropriate 3D soma/dendrite/axon morphologies, using the up-scaling routines specified in the Method section (Figures 3C–E). The somata and dendrites of each neuron were converted into 3D PST surface densities, reflecting the respective surface areas multiplied with connection-specific PST distributions. Likewise, dendrites of excitatory neurons and axons of all neurons were converted into 3D PST spine and bouton distributions, respectively (see meta-connectivity list online for all values). The resultant total soma/dendrite surface area (i.e., of all neurons in rat vS1) was 1.9 × 10^10^ μm^2^. The total number of spines was 5.2 × 10^9^, and the total number of boutons was 6.4 × 10^9^.

The average bouton (synapse) density across entire rat vS1 was 1 bouton per μm^3^, which matches previous measurements (0.94 ± 0.12 synapses per μm^3^) of synapse densities using electron-microscopic tomography on small tissue (~200 μm^3^) volumes of rat vS1 (Merchan-Perez et al., 2014). Hence, the up-scaled model of entire rat vS1 resembles the average structural organization of this brain region at mesoscopic (geometry within 50 μm inter-animal variability), microscopic (cellular distributions within 7% inter-animal variability) and nanoscopic (bouton densities) scales. Consequently, within the margins specified by the respective inter-animal variability (SDs of geometry, soma distribution, cell type-specific dendrite/axon projections, and spine/bouton densities), we consider the dense 3D model of rat vS1 as a precise average representation of this particular piece of neuronal tissue.

### Application example 2: statistical connectome of rat vS1

**Figure 4:**
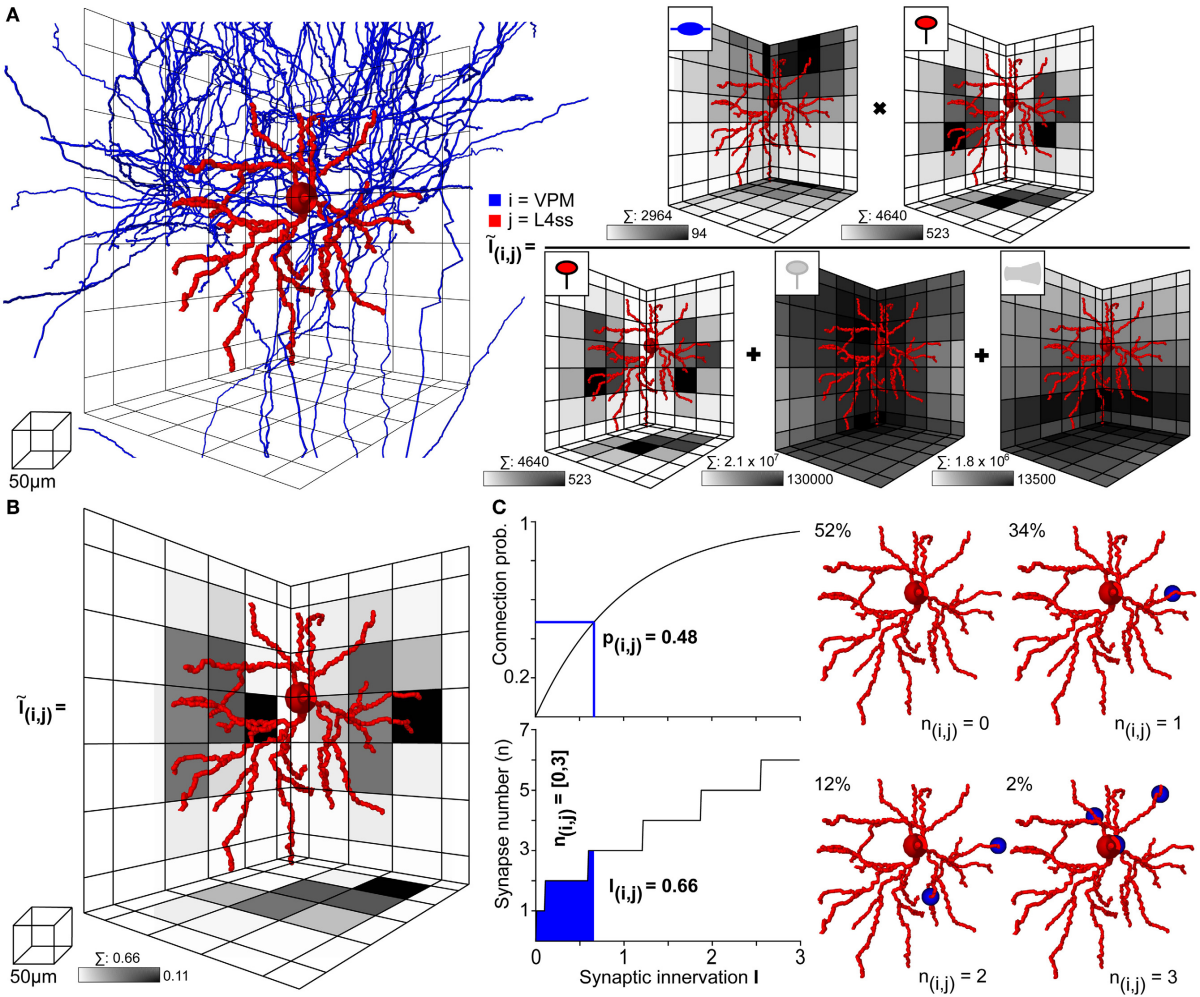
**Computation of statistical innervation between neurons in dense networks**. **(A)** Left: VPM axon (blue) and L4ss dendrite (red) from Figures 3C–E. The grid used for computing bouton, spine and dendrite surface densities is shown for scale. Right: Calculation of the 3D innervation density *Ĩ_ij_*($\vec{x}$) from the VPM axon to the L4ss dendrite. The gray-colored squares in the grid represent the maximum projection of the respective pre/postsynaptic quantity. Scale bar shows maximum value of the respective pre/postsynaptic quantity in the grid. Above each scale bar, the total number of pre/postsynaptic elements in the grid is shown. **(B)** Resulting subcellular 3D innervation density *Ĩ_ij_*($\vec{x}$). **(C)** Left top: Connection probability from neuron *i* to neuron *j* as a function of the total innervation *I_ij_*. Bottom: Possible range of the number of synapses from neuron *i* to neuron *j, n_ij_* (95th percentile for *n* > 0) as a function of the total innervation *I_ij_*. Right: Four possible synapse distributions and their probability of occurrence for the innervation from the VPM axon to the L4ss dendrite, computed from the 3D innervation density in **(B)**.

Within the dense model of rat vS1, we used *NN* to determine structural overlap of PSTs and presynaptic boutons between all pairs of neurons, always taking all neurons present in the respective overlap volumes into account. Figure 4 illustrates this process on the example of one L4ss neuron (*j*) being innervated by one thalamocortical axon (*i*) originating in VPM (Figures 3C–E). First, *NN* determines the bounding box (BB) around the dendrites of the postsynaptic neuron (Figure 4A left) and calculates the number of PSTs for each 50 μm voxel within the BB. In case of VPM neurons innervating L4ss (i.e., excitatory cell types), PSTs are limited to spines (Schoonover et al., 2014) as specified in the meta-connectivity input file (see Methods). The exemplary L4ss neuron comprises a total of 4640 spines, with a maximum of 523 spines per voxel (Figure 4A right). Second, *NN* determines the number of presynaptic boutons present in any voxel where dendrites and axons of the two neurons overlap. For the present example, the particular VPM axon has a total of 2964 boutons in the overlap volume, with up to 94 boutons per voxel.

However, within the overlap volume, dendritic spines originating from other excitatory neurons are present, rendering as equally likely contact sites for the VPM boutons as the spines of the exemplary L4ss neuron. The total number of spines within the BB of the overlap volume was 2.1 × 10^7^, with a maximum of 130,000 spines per voxel. Furthermore, VPM axons could also target somata and/or dendritic shafts of inhibitory interneurons (Staiger et al., 1996, as specified in the meta-connectivity input file), where a total of 1.8 × 10^6^ PSTs on inhibitory surfaces are present within the BB of the overlap volume, with a maximum of 13,500 surface PSTs per voxel. Consequently, the 3D innervation field *Ĩ_ij_*($\vec{x}$) between the dendrites of the L4ss neuron (*j*) and the axon of the VPM neuron (*i*), was determined with respect to all other potential PSTs (i.e., excitatory and inhibitory) present in the overlap volume. In addition, the number of all available target sites (2.3 × 10^7^) was four orders of magnitude larger than the number of spines/boutons from the individual neurons, justifying the approximation of the binomial connection probability by a Poisson distribution.

The resultant 3D innervation field *Ĩ_ij_*($\vec{x}$) between the two exemplary neurons is shown in Figure 4B. Summing across all voxels results in the total innervation *I_ij_* = 0.66, with a maximal innervation of 0.11 per voxel. This innervation number corresponds to a pairwise connection probability of *p_ij_* = 0.48, and to a range of putative synapses between *i* and *j* of *n_ij_* = 0–3 (Figure 4C left). Thus, even though the axonal arbor of the example VPM neuron displays substantial overlap with the dendritic arbor of the example L4ss neuron, the probability of these two neurons being connected according to our quantitative implementation of Peters' rule is less than 50%. Because there are on the order of 1000 other potential postsynaptic target neurons projecting dendrites into the overlap region, approaches that calculate connectivity from structural overlap without normalization by the total number of PSTs (e.g., Brown and Hestrin, 2009) will result in gross overestimation of connection probabilities.

In consequence, we argue that structural axo-dendritic overlap should never be calculated from sparse morphological data alone and that connectivity measurements by Peters' rule should not be presented in a binary fashion (i.e., overlap equals connectivity, no overlap equals no connectivity). Instead, structural overlap in the present form results in innervation measurements at subcellular (reference frame) resolution, which can be converted into pairwise connection probabilities and a range of putative synapse numbers. In case of the present example, the overlap between 2964 VPM boutons with 4640 L4ss spines did thus not result in a connection probability of 1, but instead, the probability that the two neurons were unconnected was 52%, that they were connected by a single synapse was 34%, and by two or three synapses was 12% and 2%, respectively (Figure 4C right).

### Application example 3: comparison of *in silico* with *in vitro/vivo* connectivity

**Figure 5:**
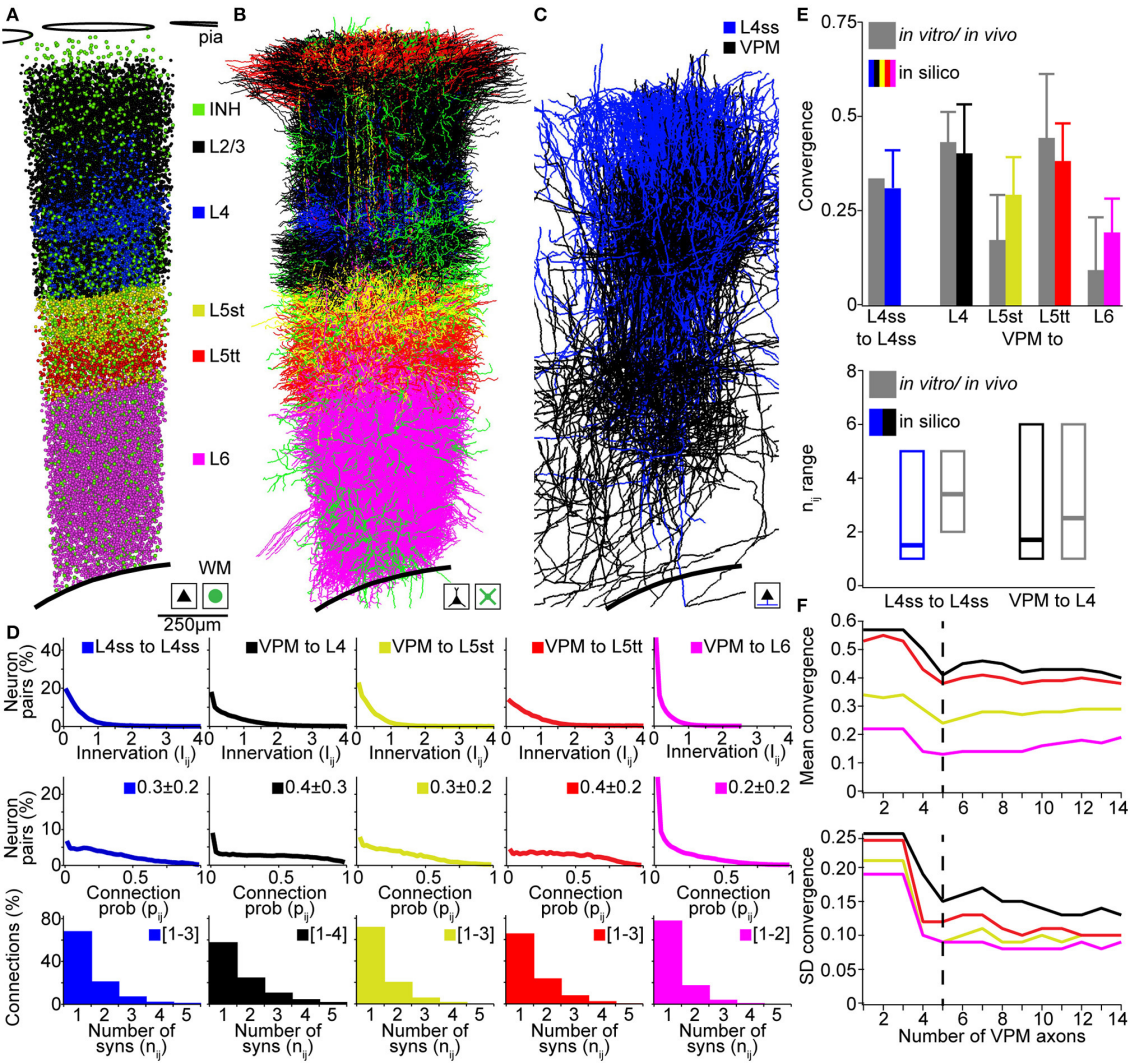
**Validation of the rat vibrissal cortex statistical connectome**. **(A)** Cell type-specific distribution of neuron somata in the model D2 column. **(B)** Cell type-specific distribution of dendrites in the model D2 column from **(A)**. Note that large basal dendrites of L3 pyramidal neurons located in the septum around the L4 barrel obscure dendrites of L4ss located inside the barrel. **(C)** Distribution of L4ss axons (blue) and VPM axons (black) in the model D2 column from **(A)**. **(D)** Distribution of neuron-to-neuron innervation *I_ij_*, the neuron-to-neuron connection probability *p_ij_* and the average distribution of the number of synapses per connection *n_ij_* for the four postsynaptic cell types in **(B)** and the two presynaptic cell types in **(C)**. **(E)** Comparison of pair-wise connectivity statistics in the model D2 column (*in silico*) and experimental results from physiological and anatomical measurements *in vitro* and *in vivo*. Top: convergence of intra-barrel connectivity and thalamocortical connectivity from VPM. Bottom: Observed and calculated range of number of synapses per connection (*in silico*: 99% cumulative range of the average distribution of *n_ij_*). **(F)** Effect of the size of the sparse morphological sample on connectivity measurements. Top: Mean convergence of thalamocortical input from VPM to four cell types in the model D2 column (see **E** for color-code) as a function of the VPM axon sample size. Bottom: Standard deviation of the convergence of thalamocortical input to these four cell types as a function of the VPM axon sample size.

In the following, we compare our *in silico* measurements of pairwise connection probabilities and putative synaptic contact sites with previously reported measurements in rat vS1 using (i) paired recording/reconstruction between L4ss neurons *in vitro* (Feldmeyer et al., 1999; Petersen and Sakmann, 2000), (ii) dual recordings and correlation analysis between VPM and L4, L5A, L5B, and L6 neurons *in vivo* (Bruno and Sakmann, 2006; Constantinople and Bruno, 2013), and (iii) electron-microscopic reconstructions of synaptic contact sites between VPM and individual L4ss neurons (Schoonover et al., 2014). For comparison, we restricted *in silico* connectivity measurements between the respective cell types to neurons located within a single barrel column (D2, Figures 5A–C) and averaged connectivity measurements across all neurons of the respective D2 populations.

The D2 column comprised 17810 excitatory neurons including 4657 neurons of L4 cell types (2480 L4ss; 1707 L4sp; 470 L4py), 1386 L5st, 1103 L5tt, 1391 L6cc, 767 L6inv, and 4048 L6ct neurons. Further, the D2 column model contained 2545 inhibitory neurons and 311 thalamocortical axons originating in the D2 barreloid (Meyer et al., 2013) of the VPM. Computing the innervation *I_ij_* for all pairs of VPM and L4, L5st, L5tt, and L6 neurons, respectively, as well as for all pairs of L4ss neurons, allowed calculating the respective neuron-to-neuron connection probabilities *p_ij_* and the average distribution of the number of synapses per connection *n_AB_* (Figure 5D). Further, we computed the cell type averages of (i) convergence between L4ss neurons, as well as between VPM and L4, L5st, L5tt, and L6 neurons in our D2 column model, and (ii) the 99th percentile of the number of putative synapses, and compared these numbers to experimental results (Figure 5E). The *in silico* L4ss-to-L4ss convergence measurements yielded a value of 0.31 ± 0.10, compared to 0.31–0.36 as measured *in vitro* (Feldmeyer et al., 1999; Petersen and Sakmann, 2000). VPM-to-L4 convergence was 0.40 ± 0.13 (*in silico*), compared to 0.43 ± 0.08 (*in vivo*). VPM-to-L5st convergence was 0.29 ± 0.10 (*in silico*), compared to 0.17 ± 0.12 (*in vivo*). VPM-to-L5tt convergence was 0.38 ± 0.10 (*in silico*), compared to 0.44 ± 0.17 (*in vivo*) and VPM-to-L6 convergence was 0.19 ± 0.09 (*in silico*), compared to 0.09 ± 0.14 (*in vivo*) (Bruno and Sakmann, 2006; Constantinople and Bruno, 2013). The *in silico* measurements of pair-wise connection probabilities matched the previously reported cell type-specific values within one SD. Interestingly, even though somata of the different cell types intermingled within and across cortical layers, our model predicted cell type-specific differences in synaptic connectivity within layers (e.g., VPM to L5st vs. L5tt). These findings are in line with previous reports that revealed that synaptic connectivity is in general cell type- and not layer-specific (Shepherd et al., 2005; Brown and Hestrin, 2009). To further evaluate how the sample size of morphological reconstructions affects our connectivity estimates, we repeated these measurements and progressively increased the number of VPM axons used for up-scaling from 1 to 14. We found that increasing the sample size beyond ~5 VPM axons did not change our results (Figure 5F), indicating that at least 5 axon reconstructions are required to capture the variability of projection patterns (at 50 μm resolution) within a cell type.

Finally, the range of putative synapses per connection for L4ss-to-L4ss connections was 1–5 (*in silico*), compared to 2–5 (*in vitro*, Feldmeyer et al., 1999). For VPM-to-L4 connections, the range was 1–6 (*in silico*), compared to 1–6 (*in vivo*, Schoonover et al., 2014). Whereas the *in silico* ranges of putative synapses per connection matched the previous *in vitro/vivo* results, our predictions showed that the most likely scenario for interconnected L4ss should be that they share only a single synaptic connection. However, reconstructions from paired-recordings revealed a more bimodal distribution, i.e., pairs of L4ss share either no contacts, or if they are connected, they share more than one contact (Feldmeyer et al., 1999). This potential discrepancy could arise from limitations to identify weakly connected L4ss (i.e., just one synaptic contact) using paired-recordings, or could indicate that our assumption of independent synapse formation is not justified for L4ss.

### Application example 4: analysis of higher-order connectivity patterns

Because the average dense model of rat vS1 resembles the structural organization of this neuronal tissue at meso-, micro- and nanoscopic scales (see Application example 1) and structural overlap measurements within the model reproduced cell type-specific pairwise connectivity measurements (see Application example 3), we investigated whether the resultant dense statistical connectome can be used to investigate higher-order connectivity patterns beyond pairwise measurements.

**Figure 6:**
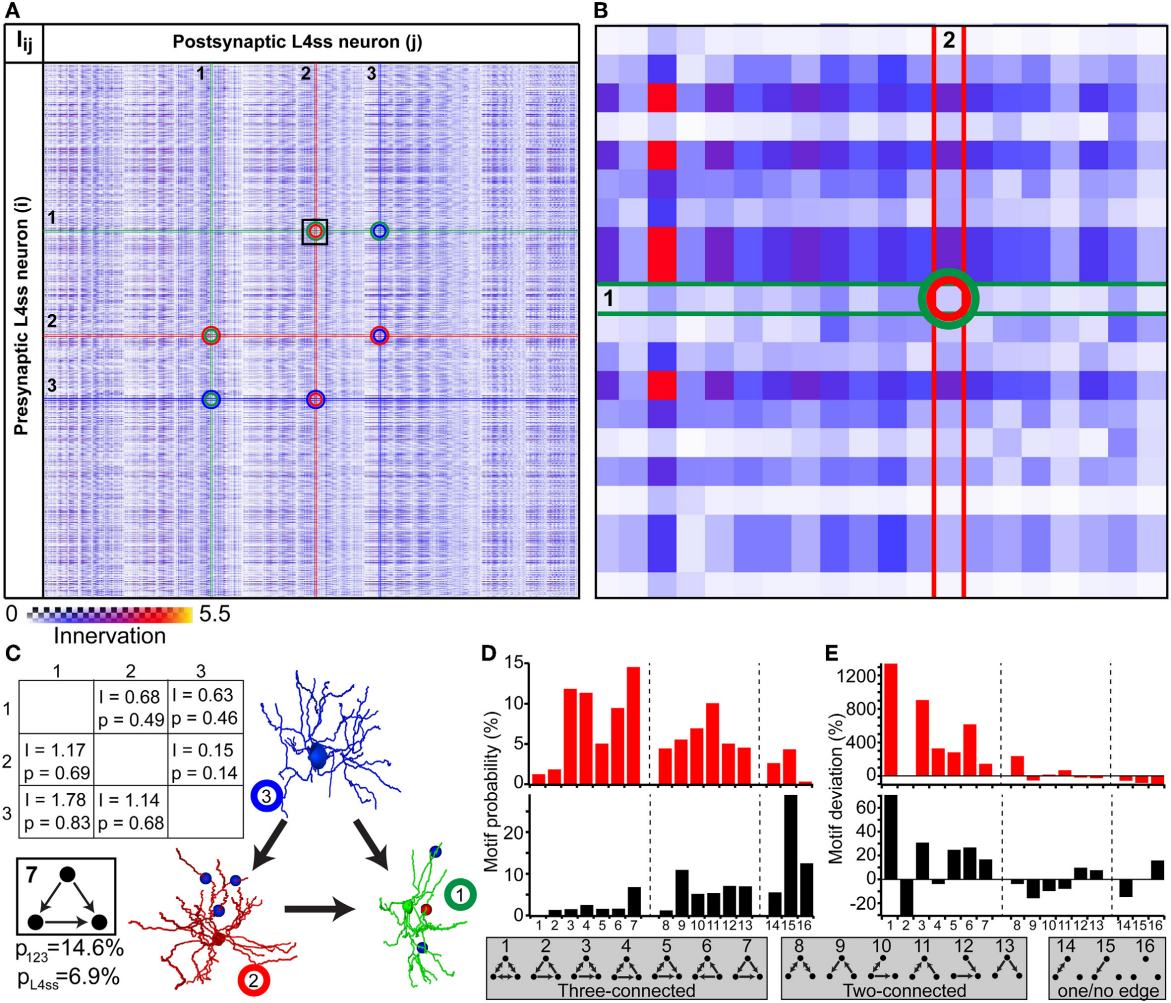
**Higher-order connectivity in dense statistical connectomes**. **(A)** The connection matrix between L4ss neurons of the D2 barrel in rat vibrissal cortex. Each entry represents the innervation *I_ij_* between pre- and postsynaptic neurons *i* and *j*. Connections between three neurons are highlighted. **(B)** Zoom into the connection matrix (see box in **A**) around the matrix entry representing the connection from neuron 1 to neuron 2. **(C)** Left: Innervation between three example L4ss neurons (highlighted in **A**), and the respective connection probabilities and strengths (see also Figure 4C). Right: One possible configuration of a three-neuron motif between these three neurons. Bottom: Summation over all configurations resulting in this motif (motif ID 7) gives the total probability of occurrence of this motif for these three neurons and the L4ss network, respectively. **(D)** Probability of finding each non-redundant three-neuron motif, calculated from the pairwise innervation. All 16 non-redundant motifs are listed at the bottom. Top: Motif distribution for the three neurons from **(C)**. Bottom: Motif distribution for the L4ss network from **(A)**. **(E)** Deviation of motif occurrence probability from expected value based on the average connection probability of L4ss neurons. Top: Three neurons from **(C)**. Bottom: L4ss network from **(A)**.

The simplest higher-order pattern to be investigated is connectivity between three neurons (Sporns and Kotter, 2004; Song et al., 2005), in the following referred to as triplet motifs. To do so, we calculated the innervation matrix *I_ij_* (i.e., dense statistical connectome) for the population of L4ss neurons within the D2 barrel column and randomly selected three neurons from the matrix (Figures 6A–B). The six entries specifying innervation between the three neurons in the *I_ij_* matrix yield connectivity statistics about each possible connection in terms of triplet motifs. Triplet motifs are illustrated as triangles of nodes (i.e., each node representing one of the three respective neurons, Figure 6C), connected by uni- and/or bidirectional edges (i.e., each edge representing synaptic connections between two neurons, and the direction specifies pre- and postsynaptic partners, respectively). For example, the innervation from neuron 1 to neuron 2 is determined by the matrix entry *I*_12_ = 0.68, which corresponds to a pairwise connection probability of *p*_12_ = 0.49. This can be interpreted as the probability that the triplet motif contains an edge from node 1 to node 2. Conversely, the probability that this particular edge is missing is 1-*p*_12_ = 0.51.

In general, three nodes can be connected by 64 different motifs of bidirectional edges. However, multiple motifs are redundant (e.g., 1 connected to 2 and no other edge present is the same motif as 2 connected to 3 and no other edge is present). Thus, the 64 triplet motifs can be reduced to 16, of which 7 contain three edges (three-connected), 6 contain two edges (two-connected, 2 contain one edge (one-connected) and 1 motif (no edges) represents the absence of any connections between the three neurons (Figures 6D–E). Using the pairwise connection probabilities for the three example neurons (i.e., *p*_12_, *p*_21_, *p*_13_, *p*_31_, *p*_23_, *p*_32_) allows computing the probability of finding each triplet motif by multiplying the probability of finding/not finding all six possible edges. For example, the probability that the three neurons are connected according to motif 7 (i.e., three-connected by unidirectional edges) is computed as follows:

$$p=(1-p_{12})\cdot p_{21}\cdot (1-p_{13})\cdot p_{31}\cdot (1-p_{23})\cdot p_{32}=0.092$$

There are five other possibilities of arranging connections between these three neurons that result in the same triplet motif. Thus, the total probability of finding this triplet motif among these three neurons is the sum over these six individual connection arrangements, resulting in a total probability of *p*_123_ = 0.146 (Figure 6C).

In the same way, we calculated the probability of occurrence for each of the 16 possible non-redundant triplet motifs, illustrated as a motif spectrum (Sporns and Kotter, 2004, Figure 6D top). Further, we extended the motif analysis to the entire population of L4ss neurons in the D2 model, by repeating the motif probability calculations 10 times for 2000 randomly selected neuron triplets. Each triplet was allowed to share at most one neuron with any other triplet. For each triplet, we computed the motif spectrum as described for the example neurons, and averaged these spectra to obtain the distribution of triplet motifs within the L4ss network (Figure 6D bottom). Finally, we compared the triplet motif spectrum of the L4ss network in a D2 barrel column with the distribution expected when assuming uniform connectivity. This scenario represents the case where average pairwise connection probabilities are known (e.g., *p* = 0.31 between L4ss neurons, as determined statistically or by paired recordings) and connectivity within the population is assumed to be homogenous (i.e., lack of variability within a population caused by cell- and/or location-specific morphological variations).

The deviations between the “uniform” spectra of triplet motifs from those predicted by the dense statistical connectome were substantial (Figure 6E). For example, motif 2 (unidirectional loop) is much less likely (~30%) compared to assuming uniform connectivity, whereas the remaining three-connected motifs are in general more likely. In contrast, two-connected motifs are in general less likely. Thus, the average dense model of the L4ss network yields high-order connectivity patterns that are significantly (*p* < 0.0001, z-score > 5 for all motifs except for motifs 8 and 15) different from a uniformly connected random network with equal pairwise connection probability.

## Discussion

In the present study, we introduced a novel quantitative approach for measuring synaptic connectivity at subcellular resolution and mesoscopic scales. The measurements are based on sparse morphological datasets, integrated into a common anatomical reference frame that allows up-scaling to an average dense model of the neuronal circuitry and determining axo-dendritic overlap between any two neurons in the model. Illustrating our approach for excitatory thalamo- and intracortical circuits in rat vS1, we (i) defined the mandatory anatomical information required to generate average dense circuit models, (ii) introduced the interactive software environment *NN* to calculate Peters' rule with respect to all neurons present in axo-dendritic overlap volumes, and (iii) found that our cell type-specific *in silico* measurements are in line with previously reported *in vitro/vivo* data.

### Previous approaches to generate average neuronal network models

In recent years, multiple approaches began integrating morphological data to generate anatomically well-constrained neuronal network models. However, compared to *NN*, where synaptic connectivity is measured within the circuit model itself, previous approaches require synaptic connectivity data as input. For example, *neuroConstruct* (Gleeson et al., 2007) connects randomly distributed neurons to networks using average pairwise connection probabilities, thereby neglecting for example location-specific differences in connectivity. *BlueBuilder* (Kozloski et al., 2008), developed within the BlueBrainProject (Markram, 2006), generates neuronal networks, where *in vitro* labeled dendrite and axon morphologies are integrated into an idealized cortical column (i.e., neglecting column-specific geometry and soma distributions) and putative dendrite-axon contacts (at a predefined distance) are pruned until they match predefined connectivity statistics (originating from paired-recordings *in vitro*, Ramaswamy et al., 2012).

Therefore, we argue that our approach can be regarded as more general for investigating structural organization principles of the neuronal circuitry. First, the present concept relies on definition of a standardized 3D reference frame that describes the average geometry of the brain structure (and substructures) of interest. Consequently, no assumptions about the mesoscopic organization of neuronal circuits are required. For example, in case of rat vS1, we previously reported that each cortical barrel column has a specific diameter, height and orientation, and barrel columns representing whiskers located within different rows along the animals' snout have substantially deviating volumes (Egger et al., 2012). Such whisker row-specific organization patterns may substantially influence connectivity, e.g., increased connectivity between columns in the same row compared to across whisker rows, an effect that would be missed by assuming that cortical columns are elementary and uniform structural building blocks (Markram, 2006).

Second, the up-scaling to a dense average circuit model is based on measured 3D distributions of excitatory and inhibitory neurons. Consequently, no assumptions about the microscopic (i.e., cellular) organization of the neuronal circuits are required. For example, in case of rat vS1, we previously reported that separation between individual barrel columns is only present within the distribution of excitatory neurons in L4, where neuron densities are significantly lower in the septum, compared to densities in barrel columns (Meyer et al., 2013). In contrast, neither excitatory distributions in superficial and infragranular layers, nor densities of inhibitory somata throughout the cortical sheet displayed differences between columns and septa. Such excitatory/inhibitory location-specific cellular organization patterns may substantially influence connectivity, e.g., the relative fraction of excitatory to inhibitory connections may be higher within the L4 barrel compared to septa and/or other layers (van Vreeswijk and Sompolinsky, 1996), effects that would be missed by assuming uniform and/or randomly distributed neuron somata (Rockel et al., 1980; Carlo and Stevens, 2013).

Finally, connectivity measurements are based upon complete 3D reconstructions of *in vivo* labeled neurons. Consequently, no assumptions about (sub)cellular organization of the neuronal circuits are required. For example, in case of rat vS1, we previously reported that axons of excitatory neurons are in general not confined to the dimensions of a single cortical column (Oberlaender et al., 2011). Thus, extrapolation of dendrite/axon morphologies from *in vitro* labeling/reconstruction (Hill et al., 2012; Ramaswamy et al., 2012) will miss cell type and/or location-specific horizontal axonal projection patterns, resulting in assessments of connectivity by structural overlap that are biased toward close-by neurons (e.g., within columns compared to across columns). Further, substantial cutting of dendrites/axons during multi-electrode recordings *in vitro* will result in unsystematically hampered measurements of pairwise connection probabilities (i.e., depending on cell type, location and distance of the recorded neurons), questioning whether constraining connectivity within neuronal network models by such data (Lefort et al., 2009; Perin et al., 2011) will result in anatomically realistic representations of the neuronal circuitry.

In summary, because organizational principles of the neuronal circuitry are generally influenced by brain region- and species-specific mesoscopic, cellular and subcellular quantities, generation of well-constrained network models should not be based on assumptions, but on measurements of these quantities instead. Assessments of these quantities provide information about the respective variability across animals, allowing to determine (i) the appropriate resolution for connectivity measurements within an average representation of the neuronal circuitry and (ii) how representative the average model is (i.e., in terms of SDs of (sub)cellular properties).

### Validity of peter's rule

The validity of measuring synaptic innervation by structural overlap between dendrites and axons has been discussed controversially (Stepanyants and Chklovskii, 2005; Shepherd et al., 2005; Mishchenko et al., 2010; Briggman et al., 2011). Specifically, reconstructions at electron-microscopic resolution provided evidence that proximity of axons and dendrites at submicron resolution in general does not imply that the two neurons form synaptic contacts (Mishchenko et al., 2010). Further, pairwise connection probabilities obtained by paired-recordings *in vitro* were considered to contradict measurements of structural overlap after reconstructing morphologies of the respective neuron pairs (Shepherd et al., 2005; Brown and Hestrin, 2009).

However, to date, neither the appropriate spatial resolution to apply Peters' rule, nor a coherent framework to obtain structural overlap in terms of connection probabilities with respect to all neurons projecting dendrites into the overlapping volume existed. We provide both. First, the resolution for determining structural overlap within an average network model (i.e., integration of morphological data from different animals) is defined by the inter-animal variability of the geometrical reference frame used to integrate the data. Increasing the voxel size will provide less accurate connectivity estimates (i.e., cells or cell types that do not overlap at 50 μm resolution may overlap at 100 μm scales). In contrast, decreasing the voxel size below the precision of the registration framework would imply inappropriate accuracy. Hence, implications of synaptic innervation below the resolution limit, or even at submicron resolution, are beyond the limits of Peters' rule. Instead, measurements of subcellular synapse locations remain exclusive to reconstructions at electron-microscopic levels (but see, Druckmann et al., 2014; Schoonover et al., 2014).

Second, we illustrate that in general, millions of potential postsynaptic target sites (PSTs) from unstained neurons are present within the overlap volume of two stained neurons. Hence, when normalizing innervation by the total number of PSTs, the resultant innervation and pairwise connection probabilities are small. In case of the exemplary calculation between the dendrites of one L4ss and one thalamocortical VPM axon in rat vS1, overlap between ~4500 spines and ~3000 boutons did not result in a connection probability of one, but instead there is a 52% chance that the two neurons are unconnected. Hence, connectivity measurements by structural overlap have to be performed with respect to *all* neurons, for example using the present approach of generating an average dense model of the brain region of interest. Consequently, the absence of synaptic contacts at touching dendrites and axons in sparsely labeled tissue should not be regarded as a violation of Peters' rule.

### Higher-order connectivity in dense statistical and electron-microscopic connectomes

In addition to illustrating that pairwise connection probabilities determined by structural overlap are in line with measurements using conventional recording/reconstruction techniques, we provide a strategy that allows investigation of higher-order connectivity patterns within dense statistical connectomes. On the example of the population of L4ss neurons located within a barrel of rat vS1, we determined the probabilities of obtaining all possible three-neuron (triplet) motifs and compared the resultant motif spectra with those to be expected from randomly connected networks that have the same average pairwise connection probability. Interestingly, we found that the two spectra displayed significant deviations. For example, unidirectional triplets (i.e., recurrent loops) are much less likely to occur within the L4ss population compared to randomly connected networks. In contrast, other triplet configurations were significantly more likely. Arguably such deviations can be considered as evidence for specificity in the organization of the neuronal circuitry, for example caused by inhomogeneous distributions of somata (e.g., excitatory soma density decreases from the barrel center toward the borders), dendrites and axons (e.g., polar dendrite morphologies pointing toward the barrel center).

Hence, we suggest using statistical spectra of higher-order motifs as a definition of cell type-specific “structural fingerprints” for the respective neuronal circuits. Comparing these fingerprints with dense connectomes obtained at electron-microscopic resolution, will indicate whether such cell type-specific higher-order patterns can be explained by the meso- and microscopic organization of the network, or whether additional specificity originates at nanoscopic scales. In consequence, not the absence of synapses between touching dendrites/axons, but deviations of higher-order connectivity patterns observed in statistical and electron-microscopic dense connectomes should be considered as evidence for violations of statistical network organization.

## Conclusion

We present a novel concept for measuring pairwise and high-order connectivity patterns at subcellular resolution and mesoscopic scales. We provide the required software to generate average dense circuit models, to calculate structural overlap, and to convert these measurements into dense statistical connectomes. Further, we describe the anatomical data necessary to assess structural organizational principles of the neuronal circuitry without assumptions about homogeneity at meso/microscopic and subcellular scales. Given that the required anatomical data is available, we consider our approach as generalizable to other brain structures and species. This sets the stage to generate well-constrained network models that allow simulating sensory-evoked signal flow to provide unprecedented insight into the interplay between the structural organization and function of the respective local and long-range neuronal circuits.

### Conflict of interest statement

The authors declare that the research was conducted in the absence of any commercial or financial relationships that could be construed as a potential conflict of interest.
